# Risk Factors for Aspiration Pneumonia among Elderly Patients in a Community-Based Integrated Care Unit: A Retrospective Cohort Study

**DOI:** 10.3390/geriatrics6040113

**Published:** 2021-11-30

**Authors:** Isao Uno, Takaaki Kubo

**Affiliations:** 1 Sakurajyuji Hospital, 1-1-1 Mikoyokibe, Kumamoto Prefecture, Minami-ku, Kumamoto 861-4173, Japan; 2 Division of Health Sciences, Graduate School of Health Sciences, Kumamoto Health Science University, 325 Izumi-Machi, Kumamoto Prefecture, Kita-ku, Kumamoto 861-5598, Japan; kubo@kumamoto-hsu.ac.jp

**Keywords:** aspiration pneumonia, community-based integrated care unit, oral health

## Abstract

We aimed to clarify the physical factors associated with the incidence of aspiration pneumonia in a community-based integrated care unit. This retrospective cohort study included 412 patients aged 65 years or older admitted to a community-based integrated care unit. A new diagnosis of aspiration pneumonia made by the attending physician based on physical examination, imaging findings, and blood test data after 48 h of admission was considered as an incidence of aspiration pneumonia. Basic patient information, activities of daily living, swallowing function, nutritional status, cognitive function, oral health-related factors, and energy intake were retrospectively investigated. We classified the patients into a pneumonia group and a non-pneumonia group, and examined the factors associated with the development of aspiration pneumonia. The mean age was 86.9 ± 8.1 years, and the pneumonia group comprised 49 participants. Comparison between the groups showed significant differences in oral environment, denture use, cognitive functional independence measure, and discharge to home. In multivariate logistic regression analysis, oral environment (odds ratio (OR) = 0.229, 95% confidence interval (CI): 0.070–0.753, *p* = 0.015) and use of dentures (OR = 0.360, 95% CI: 0.172–0.754, *p* = 0.007) were independently associated with aspiration pneumonia. Oral care and the use of dentures may be effective in preventing aspiration pneumonia.

## 1. Introduction

The most common cause of death among Japanese people is malignant neoplasm, followed by heart disease; this has remained unchanged for decades. The other causes are cerebrovascular disease, pneumonia, and senility, which have shifted in rank every year [1]. However, aspiration pneumonia is the sixth leading cause of death, and combined pneumonia and aspiration pneumonia is the third leading cause of death. It has also been pointed out that pneumonia is a common cause of death among senile older people, and it is thought that the number of people dying from pneumonia may be higher than the statistical numbers suggest. Furthermore, a survey by the World Health Organization revealed that the number of deaths due to pneumonia is high worldwide. Lower respiratory tract infections, including pneumonia, are the fourth-leading cause of death and resulted in a reported 2.6 million deaths in 2019 [2].

The incidence of pneumonia is high among elderly people, with 75% of pneumonia patients aged over 70 years [3]. Aspiration pneumonia accounts for 86.7% of pneumonia cases in older people. The risk of aspiration is increased in the elderly population because of decreased swallowing function. Risk factors for aspiration include neurogenic diseases such as cerebrovascular disease, Parkinson’s disease, dementia, and neuromuscular disease; abnormalities of the gastroesophageal junction, such as gastroesophageal reflux disease, post-gastrectomy, and nasogastric tube feeding; tumors of the oral cavity, pharynx, and larynx; and tracheostomy [4]. Aspiration is the primary cause of aspiration pneumonia, although not all instances of aspiration result in pneumonia. The development of aspiration pneumonia depends on the balance between factors related to resistance, such as respiratory and immune functions, and invasion-related factors, such as the amount of aspiration and the bacteria involved. The incidence of aspiration pneumonia is related not only to food aspiration due to dysphagia but also the invasion of bacterial pathogens in the oral cavity [5]. Further, it has been reported that 71% of elderly patients with pneumonia develop silent aspiration while sleeping [6], and the main cause of aspiration pneumonia in older patients is thought to be silent aspiration of oral bacteria [7]. Risk factors for the incidence of aspiration pneumonia include cognitive decline [8], dysphagia [9], failure to use dentures [10], the motor component of the Functional Independence Measure (FIM) score [11], poor nutritional status [12], poor oral environment [13], reduced chewing ability [14], low albumin level [15], and tube feeding [16]. These factors increase the amount of aspiration and oral bacteria and decrease resistance to invasion. The incidence of aspiration pneumonia is associated with prolonged hospitalization and increased rates of pneumonia recurrence and mortality [17]. When aspiration pneumonia occurs during hospitalization, the mortality rate is reported to be 33.3% in hospital, while it is 50.8% within six months [18], 49% at one year, 67.1% at three years, and 76.9% at five years [19]. Therefore, identifying the risk of aspiration pneumonia during hospitalization and working toward prevention may help reduce mortality rates.

In Japan, community-based integrated care units were established in 2014, and by 2019, there were 2424 hospitals and 84,813 beds [20]. The four roles of these units are: “postacute function”, “subacute function”, “rehabilitation”, and “return to home [20]”. According to a nationwide survey, more than 80% of the patients admitted to community-based integrated care units are over 70 years old [20]. Such units play an important role in reducing admissions to acute care hospitals, rise in medical expenses, and functional decline after admission to acute care hospitals. The care provided here helps in improving the functions of hospitalized patients and ensuring their discharge within a limited period of time, as the standard time for hospitalization is 60 days and the return-to-home rate is at least 70%. Adverse events during hospitalization lead to delays in discharge. Aspiration pneumonia is one of the most common adverse events occurring among elderly patients. However, the factors associated with the incidence of aspiration pneumonia in community-based integrated care units have not yet been clarified; studying these factors may help to improve the quality of medical care for older persons.

Therefore, the purpose of this study was to investigate factors associated with the development of aspiration pneumonia among elderly inpatients in a community-based integrated care unit and to propose effective measures for preventing it.

## 2. Materials and Methods

### 2.1. Participants

In this retrospective cohort study, data were collected retrospectively from the medical records of elderly patients aged 65 years or older admitted to the community-based integrated care unit of the Sakurajyuji Hospital in Japan from April 2018 to March 2020. The exclusion criteria were as follows: patients younger than 65 years, date deficiency, and patients who required short-term hospitalization for tests and procedures. Rehabilitation was performed by a physical or occupational therapist for 60 min per day. Additionally, Patients who have been identified as having possible dysphagia or who present with dysphagia on examination at admission, such as mucus buildup or difficulty swallowing during the first meal, were evaluated by a speech-language pathologist for swallowing function. They were then provided with feeding, swallowing, and oral function exercises, and food patterns were adjusted. At the time of admission, the patients attended a conference to set their goals, and the multidisciplinary team discussed their progress and current problems every week. The nutritional status of each patient was evaluated, and individualized nutritional interventions were provided to patients who were at a high risk of malnutrition.

The sample size was calculated using the G*Power software (v3.1.9.7, Düsseldorf, Germany), with alpha error = 0.05 and power = 0.8. As a result, the required n for one group was 51. Since it was estimated that the incidence of nosocomial pneumonia in our hospital was approximately 20%, we gathered the data such that the number of subjects would be approximately 400, excluding those with missing data.

### 2.2. Measurement

The following data were collected from medical records: age, gender, height, weight at admission, body mass index (BMI), FIM, serum albumin level, Food Intake LEVEL Scale (FILS), Geriatric Nutritional Risk Index (GNRI), Mini-Mental State Examination (MMSE), number of remaining teeth, oral environment, denture usage, necessity of dental treatment, energy requirements, energy intake, energy sufficiency ratio, length of stay in the unit, diseases prescribed for rehabilitation, hospitalization circumstances, discharge destination, and occurrence of aspiration pneumonia.

A new diagnosis of aspiration pneumonia made by a physician based on clinical symptoms (fever, increased sputum volume, worsening respiratory status), new infiltrative shadows on chest radiography or computed tomography images, and blood test findings (elevated white blood cell count, elevated inflammatory response) within 48 h of admission was considered as an incidence of aspiration pneumonia. Weight at admission was measured by a nurse, and BMI was calculated at admission using weight and height measurements using the formula weight (kg)/height (m^2^). The FIM is a tool used to measure the degree of independence in daily life; each item is scored on a 7-point scale ranging from 1 point (“total assistance”) to 7 points (“independence”) [21]. It consists of 18 items, 13 related to exercise and five related to cognition. Possible scores range from 18 to 126. The FIM scores were allocated by the therapist upon admission. Motor, cognitive, and total FIM scores were assigned separately. Serum albumin level values were extracted from the blood test data at the time of admission. The FILS reflects the feeding status of patients on a 10-point scale; higher scores indicate better feeding status [22]. The FILS was assessed by a dietitian upon admission by checking dietary intake. The GNRI is a screening tool employed to evaluate the risk of malnutrition, and is calculated by using the formula (14.89 × serum albumin level) + 41.7 × (current weight/standard weight). Scores of <82, 82–91, 92–97, and ≥98 indicate severe nutritional impairment risk, moderate nutritional impairment risk, mild nutritional impairment risk, and no risk, respectively [23]. The GNRI was calculated using the height, weight, and serum albumin level measurements at the time of admission. The MMSE is a measure of cognitive function that is used in a variety of settings worldwide. It consists of eleven questions that can be assigned a maximum score of 30 points; a score of 21 or below indicates possible cognitive impairment [24]. MMSE score was assessed on admission by the therapist in charge. The number of remaining teeth, oral environment, denture use, and need for dental treatment were assessed by the dentist upon admission using an oral screening sheet. The oral screening sheet was originally created with reference to several oral screening tools. The oral environment was assessed comprehensively by a dentist based on tongue lichen and plaque and was rated as either “good”, “little bad”, or “bad”. Energy requirements were calculated using the Harris-Benedict equation. The stress factor was ascribed by a dietitian based on the report by Long et al., considering the general condition of the patients [25]. The activity factor ranging between 1.1–1.7 was assigned by the therapist in charge depending on the amount of activity in daily life and the amount of activity during each therapy session. The level of each activity performed during the day was judged as follows: low (always in bed), medium (performing only minimal activities such as rehabilitation and activities of daily living (ADL)); and high (performing activities such as self-discipline other than rehabilitation and ADL) The amount of activity performed during each therapy session was defined as follows: low (exercise in supine or seated position with assistance to automatic movement), medium (exercise in antigravity position and low-load strength training), and high (active exercise, such as walking and high-load strength training). A dietician calculated energy intake by extracting data on the percentage of food intake from the medical records of each patient for one week after admission. The energy sufficiency ratio was calculated as the ratio of energy intake to energy requirement. The number of days in the ward was defined as the number of days spent in the community-based integrated care unit. Hospitalization history and discharge destination were investigated from medical records.

### 2.3. Statistical Analysis

Depending on the incidence of aspiration pneumonia during hospitalization, the participants were categorized into the pneumonia and non-pneumonia groups. In addition, χ^2^ tests were performed for items with binary variables. Multivariate logistic regression analysis was performed, considering the incidence of aspiration pneumonia as the dependent variable. As explanatory variables, we selected items that have been shown to be associated with aspiration pneumonia in previous studies and have high clinical utility, and performed a forward-backward stepwise selection method. EZR (Saitama Medical Center, Jichi Medical University, Saitama, Japan) was used for statistical processing, and the statistical significance level was set at less than 5%.

### 2.4. Ethical Consideration

This study is a retrospective study using medical information; therefore, participants were given the opportunity to opt out and withdraw from the study. This study was approved by the Clinical Research Ethics Review Committee of the Sakurajyuji Hospital (approval number: 2020-07).

## 3. Results

The total number of hospitalized patients during the study period was 592, and after excluding those who met the exclusion criteria, there were 412 study participants (138 men, 274 women; mean age 86.9 ± 8.1 years). Among them, 49 had aspiration pneumonia. A higher percentage of participants in the non-pneumonia group used dentures and exhibited a good oral environment. The FIM scores related to defecation management, comprehension, expressiveness, problem solving, and cognitive function scores were higher in the non-pneumonia group. Further, a higher percentage of patients in the non-pneumonia group were discharged (34% vs. 16%) (Table 1).

In the multivariate logistic regression analysis, gender, oral environment, denture use, number of remaining teeth, and cognitive FIM score were selected as explanatory variables using a stepwise method. Consequently, good oral environment (OR = 0.229, 95% CI: 0.070–0.753, *p* = 0.015) and denture use (OR = 0.360, 95% CI: 0.172–0.754, *p* = 0.007) were found to be associated with a lower risk of incidence of aspiration pneumonia (Table 2).

## 4. Discussion

In this study, we investigated the physical factors associated with the risk of aspiration pneumonia during hospitalization among elderly patients admitted to a community-based integrated care unit.

The results suggest that proper oral management may be effective in preventing aspiration pneumonia. Among the oral management factors, maintaining a good oral environment and the use of dentures were particularly effective in reducing the risk of aspiration pneumonia. On the other hand, there was no association between aspiration pneumonia and the risk factors reported in previous studies, such as swallowing function, number of remaining teeth, nutritional status, ADL ability, and cognitive function.

### 4.1. Denture Use and Risk of Aspiration Pneumonia

The use of dentures reduced the risk of aspiration pneumonia in the hospital. Takeuchi et al. reported that among elderly people aged ≥70 years residing in nursing homes, those at risk of aspiration are more likely to develop pneumonia; however, using dentures reduces the risk of incident pneumonia [10]. Moreover, Manabe et al. demonstrated that, among elderly people aged ≥65 years living in the local community, those with few remaining teeth were more likely to develop pneumonia, although the use of dentures decreases this risk [8]. In our study, swallowing function and the number of remaining teeth were not associated with the development of aspiration pneumonia; nevertheless, it had a significant relationship with the use of dentures. Elderly people who do not use dentures have reduced masticatory ability [26]. Moreover, salivary secretion decreases with masticatory ability. It is possible that the masticatory ability of participants who did not use dentures decreased, which resulted in reduced saliva secretion and oral self-cleaning function. This in turn could lead to deterioration of the oral environment. Therefore, it is possible that the oral environment deteriorated in elderly people who did not use dentures, and silent aspiration of bacteria that grow in the oral cavity during the night might have led to the incidence of aspiration pneumonia.

### 4.2. Oral Environment and Risk of Aspiration Pneumonia

A good oral environment reduces the risk of aspiration pneumonia. Scannapieco, et al., reported that many institutionalized elderly people have a poor oral environment, which can cause of respiratory tract infections [27]. Another study by Nishizawa et al. showed that poor oral environment and high oral bacterial counts are associated with a higher risk of aspiration pneumonia in hospitalized patients aged over 50 years [28]. The major causes of aspiration pneumonia are the invasion of bacterial pathogens and aspiration due to dysphagia. The invasion of bacterial pathogens in the oral cavity by silent aspiration is considered to be the main cause of aspiration pneumonia in older patients, and 71% of them suffer from silent aspiration at night [7]. Similar to the findings of previous studies, we found that the oral environment was associated with aspiration pneumonia, and it is possible that aspiration pneumonia was caused by silent aspiration of oral bacteria.

### 4.3. Other Factors and Risk of Aspiration Pneumonia

Factors that have been associated with the risk of aspiration pneumonia in previous studies such as swallowing function, ADL ability, cognitive function, and nutritional status were not found to be associated with it in this study. A meta-analysis by Wierink, et al. demonstrated that dysphagia is associated with a higher risk of aspiration pneumonia in frail elderly people [9]. This study also showed that elderly people with a history of cerebrovascular disease are at particularly high risk of aspiration pneumonia. In our study, poor swallowing function and cerebrovascular disease were not associated with the development of aspiration pneumonia. Early swallowing screening by a speech-language pathologist is effective in preventing aspiration pneumonia [29]. It is possible that the patients with impaired swallowing function in this study did not develop aspiration pneumonia because their dietary-care methods were examined and eating patterns were adjusted according to their swallowing function through speech-language pathology or feeding therapy. Additionally, swallowing function is related to the functioning of the muscles involved in it, saliva production, and decreased oropharyngeal sensation [30]. A previous study has shown that the onset of aspiration pneumonia patients with impaired swallowing function can be prevented by continued oral intake of water jelly administered by a speech pathologist [31]. It is possible that the improvement of swallowing and oral functions through evaluation and swallowing training by a speech-language pathologist for patients with impaired swallowing function and the ability to continue oral intake might have been a factor in preventing the onset of aspiration pneumonia.

Regarding ADL ability, Mitani, et al., reported that a low FIM motor score is associated with a higher risk of incident aspiration pneumonia among hospitalized patients [11]. However, in this study FIM motor scores did not differ between the pneumonia and non-pneumonia groups and were not associated with the risk of aspiration pneumonia. Williams et al. reported that higher physical activity is related to a lower risk of aspiration pneumonia [32]. Head-up positions, such as sitting and standing, are also considered effective in preventing aspiration pneumonia [16]. The subjects in this study started rehabilitation within 24 h if their hospitalization was on a weekday, and within 48 h if it was on a weekend. Getting them out of bed early during their hospitalization and increasing their activity level might have reduced the risk of aspiration pneumonia.

Regarding cognitive function, Naruishi, et al., reported that cognitive decline is a risk factor for aspiration pneumonia among elderly hospitalized patients [33]. Further, patients with declining cognitive function are more likely to have a deteriorating oral environment due to factors such as loss of interest in their own personal appearance, lack of understanding of how to use items, and reduced hand dexterity [34]. In this study, cognitive function as measured by the MMSE was not associated with aspiration pneumonia, although the participants in the pneumonia group scored lower than those in the non-pneumonia group on the FIM cognitive items of comprehension, representation, and problem solving. The MMSE is a cognitive function test that evaluates disorientation and memory ability. The FIM cognitive items that showed a difference between the groups in this study were related to communication ability. A decline in communication skills makes conveying information about physical conditions and subjective symptoms and listening to and understanding the cautions regarding daily life activities more challenging. Therefore, the pneumonia group participants might have had lower scores because they were unable to communicate subjective symptoms, such as difficulty in swallowing and eating and general lethargy, which may be signs of aspiration pneumonia. Further, they might have had difficulty in understanding the precautions to prevent aspiration pneumonia involving oral care and food intake.

Regarding nutritional status, Mitani, et al. reported that GNRI, a measure of nutritional status, is associated with the risk of aspiration pneumonia among elderly patients admitted to a long-term care unit [12]. In this study, GNRI was not associated with the risk of developing aspiration pneumonia. Nutritional status can be improved by aggressive nutritional therapy by considering the energy stores of patients with low nutrition [35]. In addition, intervention by the nutrition support team is effective in improving the nutritional status of hospitalized patients [36]. In our study, for patients with malnutrition or those at risk of malnutrition, multidisciplinary conferences were conducted and nutrition therapy was tailored to individual patients. It is unclear whether nutritional status improved; however, it is possible that improved nutritional status might have reduced the risk of developing aspiration pneumonia in patients with malnutrition.

### 4.4. Limitations

This study has several limitations. First, some data were missing owing to the retrospective nature of the study; therefore, the data may not be representative of the entire population of elderly inpatients in targeted community-based integrated care units. Second, factors such as physical and respiratory functions that may be associated with aspiration pneumonia were not measured because the data were collected retrospectively and could not be assessed. Physical function is an indicator of frailty and sarcopenia, and may be related to aspiration pneumonia. Respiratory function is indicative of the ability to expel sputum and foreign substances, and may be associated with aspiration pneumonia. However, ADL and swallowing function were not associated with aspiration pneumonia. Thus, the influence of physical and respiratory functions on our results is likely to be small. In addition, we were only able to investigate denture use. Other known risk factors for aspiration pneumonia, such as the denture nighttime wearing status and denture cleanliness, should be examined in future surveys. Third, this study was conducted in a single facility; therefore, the results cannot be generalized. The medical services provided in this facility may not necessarily be the same as those provided in other facilities, with possible differences in the medical policy of the physicians, the period of time before the start of rehabilitation, the amount of rehabilitation, the method of nutritional intervention, and the degree of multidisciplinary cooperation. Fourth, we were unable to investigate some of the other risk factors associated with aspiration pneumonia, such as inflammatory markers, disease severity, and comorbidities. Since aspiration pneumonia poses a higher risk when the body’s defenses are compromised, future studies should consider these indicators.

## 5. Conclusions

The results of this study showed that a good oral environment and the use of dentures were associated with a reduced risk of aspiration pneumonia among elderly inpatients in a community-based integrated care unit. However, while previous studies have shown that swallowing function, ADL ability, cognitive function, and nutritional status are associated with aspiration pneumonia, our findings did not reveal such an association. Even patients exhibiting conventional risk factors for aspiration pneumonia might not have developed it because of the medical services provided. Nevertheless, we found that oral environment and denture use are risk factors for aspiration pneumonia, as exhibited in previous studies. Therefore, it is necessary that not only dentists but also other medical professionals pay attention to oral problems and consider countermeasures in order to prevent aspiration pneumonia.

## Figures and Tables

**Table 1:** Participant characteristics and comparison between groups.

	Total (*n* = 412)	Pneumonia (*n* = 49)	Non-Pneumonia (*n* = 363)	*p* Value
Age (years)	86.9 ± 8.1	86.8 ± 8.2	87.2 ± 7.7	0.778
Gender (*n*, %)				
Male	138 (34)	14 (29)	124 (34)	0.520
Female	274 (66)	35 (71)	239 (66)	
Body mass index (kg/m^2^)	19.9 ± 3.6	19.9 ± 3.9	19.9 ± 3.6	0.931
Serum albumin level (g/dL)	3.31 ± 0.5	3.24 ± 0.5	3.31 ± 0.5	0.362
Geriatric nutritional risk index (score)	91.8 ± 11.8	90.8 ± 11.5	92.0 ± 11.9	0.520
Food intake level scale (score)	10 (7–10)	10 (6–10)	10 (7–10)	0.629
Tube feeding (*n*, %)	41 (10)	6 (12)	35 (10)	0.609
Energy intake (kcal)	1183.9 ± 408.7	1205.1 ± 335.3	1181.0 ± 417.9	0.699
Energy sufficiency ratio (%)	77.5 ± 25.1	79.6 ± 23.5	77.2 ± 25.4	0.516
Mini mental state examination (score)	15.2 ± 10.4	13.4 ± 10.5	15.4 ± 10.4	0.192
Oral environment (*n*, %)				
Good	111 (27)	7 (14)	102 (28)	0.040 *
Little bad	232 (56)	29 (59)	203 (56)	0.759
Bad	71 (17)	13 (27)	58 (16)	0.710
Need for treatment (*n*, %)				
Yes	222 (54)	31 (63)	191 (53)	0.172
No	190 (46)	18 (37)	172 (47)	
Denture used (*n*, %)				
Yes	213 (52)	17 (35)	196 (54)	0.016 *
No	199 (48)	32 (65)	167 (46)	
Remaining tooth (number)	12.1 ± 10.0	12.0 ± 9.7	12.1 ± 10.0	0.956
Functional independence measure (score)				
Motor FIM	42.7 ± 24.8	37.6 ± 24.9	43.4 ± 24.7	0.125
Eating	6 (2–5)	5 (1–7)	6 (2–7)	0.208
Grooming	4 (1–6)	3 (1–5)	4 (1–6)	0.059
Bathing	2 (1–4)	1 (1–4)	2.5 (1–4)	0.152
Dressing upper	3 (1–5)	2 (1–5)	3 (1–5)	0.168
Dressing lower	3 (1–5)	1 (1–5)	3 (1–5)	0.135
Excretion	3 (1–6)	1 (1–5)	3 (1–6)	0.071
Urination	4 (1–7)	1 (1–6)	4 (1–7)	0.097
Defecation	4 (1–7)	1 (1–6)	4 (1–7)	0.048 *
Transferring bed	4 (1–6)	3 (1–5)	4 (2–6)	0.102
Transferring toilet	4 (1–6)	2 (1–5)	4 (1–6)	0.075
Transferring bath	1 (1–4)	1 (1–3)	1 (1–4)	0.318
Gait	1 (1–5)	1 (1–4)	1 (1–5)	0.267
Stairs	1 (1–1)	1 (1–1)	1 (1–1)	0.505
Cognitive FIM	22.0 ± 10.4	19.2 ± 10.0	22.4 ± 10.4	0.044 *
Understanding	5 (3–7)	4 (3–6)	5 (3–7)	0.041 *
Expression	6 (3–7)	4 (3–7)	6 (3–7)	0.030 *
Interaction	5 (3–7)	5 (1–7)	5 (3–7)	0.130
Problem-solving	4 (1–6)	3 (1–5)	4 (1–6)	0.037 *
Memory	3 (1–6)	3 (1–5)	4 (1–6)	0.099
Total FIM	64.7 ± 33.4	56.8 ± 33.0	65.7 ± 33.4	0.078
Disease (*n*, %)				
Locomotor	124 (30)	19 (39)	105 (29)	0.184
Respiratory	87 (21)	7 (14)	80 (22)	0.264
Cerebrovascular	42 (10)	3 (6)	39 (11)	0.451
Cardiovascular	32 (8)	3 (6)	29 (8)	1.000
Disuse	127 (31)	17 (35)	110 (30)	0.515
Speech therapy	99 (24)	14 (29)	85 (23)	0.476
Feeding therapy	60 (15)	9 (18)	51 (14)	0.394
Admission (*n*, %)				
Outpatient	150 (36)	18 (37)	132 (36)	1.000
Clinic	57 (14)	5 (10)	52 (14)	0.516
Acute hospital	114 (28)	11 (22)	103 (28)	0.496
Other hospitals	26 (6)	3 (6)	23 (6)	1.000
General unit	55 (13)	11 (22)	44 (12)	0.070
Long term care unit	6 (1)	0 (0)	6 (2)	1.000
Rehabilitation unit	4 (1)	1 (2)	3 (1)	1.000
Discharge (*n*, %)				
Home	132 (32)	8 (16)	124 (34)	0.014 *
Nursing home	149 (36)	24 (49)	125 (34)	0.057
Other facilities	24 (6)	3 (6)	21 (6)	1.000
Acute hospital	11 (3)	1 (2)	10 (3)	1.000
Other hospitals	6 (1)	1 (2)	5 (1)	1.000
General unit	35 (8)	5 (10)	30 (8)	0.590
Long term care unit	42 (10)	4 (8)	38 (10)	0.803
Rehabilitation unit	4 (1)	2 (4)	2 (1)	0.071
Death	9 (2)	1 (2)	8 (2)	1.000

Median (minimum—maximum). Mean ± SD. FIM: Functional independence measure. * Significantly different from pneumonia (*p* < 0.05).

**Table 2:** Multivariate logistic regression analysis with aspiration pneumonia as dependent variable.

	Odds Ratio	95% Confidence Interval	*p* Value
Male	2.26	0.872–5.880	0.094
Good oral environment	0.229	0.070–0.753	0.015 *
Denture used	0.360	0.172–0.754	0.007 **
Remaining tooth	0.965	0.931–1.000	0.056
Cognitive FIM ^a^	0.974	0.946–1.000	0.070

FIM: functional independence measure. * *p* < 0.05, ** *p* < 0.01.

## Data Availability

The data presented in this study are available on request from the corresponding author.
